# Value of systemic inflammation markers for the detection of minimal and prediction of overt hepatic encephalopathy after TIPS insertion

**DOI:** 10.1007/s11011-024-01436-2

**Published:** 2024-12-10

**Authors:** Anja Tiede, Lena Stockhoff, Alena F. Ehrenbauer, Hannah Rieland, Markus Cornberg, Bernhard C. Meyer, Maria M. Gabriel, Heiner Wedemeyer, Jan B. Hinrichs, Karin Weissenborn, Christine S. Falk, Benjamin Maasoumy

**Affiliations:** 1 Department of Gastroenterology, Hepatology, Infectious Diseases and Endocrinology, Hannover Medical School, Hannover, Germany; 2 German Center for Infection Research (DZIF), Hannover-Braunschweig, Germany; 3 Center for Individualized Infection Medicine (CIIM), c/o CRC Hannover, Hannover, Germany; 4 Department of Diagnostic and Interventional Radiology, Hannover Medical School, Hannover, Germany; 5 St. Bernward Hospital, Radiology, Hildesheim, Germany; 6 Department of Neurology, Hannover Medical School, Hannover, Germany; 7 Institute of Transplant Immunology, Hannover Medical School, Hannover, Germany

**Keywords:** TIPS (Transjugular Intrahepatic Portosystemic Shunt), Systemic Inflammation, mHE (Minimal Hepatic Encephalopathy), PHES (Psychometric Hepatic Encephalopathy Score)

## Abstract

Development of overt hepatic encephalopathy (oHE) is a particularly feared complication when considering treatment with transjugular intrahepatic portosystemic shunt (TIPS). However, the pathophysiology of HE, in particular after TIPS-insertion, is complex and valid predictors remain scarce. We aimed to investigate whether systemic inflammation markers (SIM) are linked to minimal (mHE) and overt HE (oHE) development before and after TIPS. 62 prospectively recruited patients undergoing TIPS-insertion were included and monitored for oHE occurrence two years thereafter. Patients underwent psychometric testing including the portosystemic encephalopathy syndrome test (PSE), yielding the psychometric hepatic encephalopathy score (PHES), and Animal Naming Test (ANT) before TIPS (baseline) and during structured follow-up 1, 3, 6 and 12 months afterwards. SIM (IL-6, TNF-α and IL-1β) were measured at corresponding timepoints. Patients were predominantly male (64.5%) with a median age of 58 years and MELD of 11. The majority (75.8%) received a TIPS for treatment of refractory ascites. 67.9% presented with mHE before TIPS. No link between the investigated SIM and PHES or ANT at baseline or during any follow-up was documented. 19 (30.6%) patients developed oHE during follow-up. Neither baseline SIM levels nor test results were significantly associated with risk for oHE. We demonstrated a significant decline of all SIM during follow-up, which did not translate to an ameliorated risk for oHE. In patients undergoing TIPS-insertion, the selected SIM have neither a strong link to post-TIPS-oHE development nor to subclinical changes in psychometric tests for mHE.

**Supplementary Information:**

The online version contains supplementary material available at 10.1007/s11011-024-01436-2.

## Introduction

In the natural history of liver cirrhosis, the onset and progression of portal hypertension is widely considered the main driver of cirrhosis-related complications (D’Amico 2006; de Franchis et al. 2022). The prognosis of patients markedly decreases after developing a decompensating event, most commonly ascites or variceal bleeding (European Association for the Study of the Liver 2018; D’Amico et al. 2018; de Franchis et al. 2022). An established causal treatment for these portal hypertension related complications is the insertion of a transjugular intrahepatic portosystemic shunt (TIPS). While TIPS has been shown to decrease the risk for hepatic decompensation, including variceal bleeding and ascites (Larrue et al. 2023), other complications can arise. One of the most frequent complications after TIPS is the onset of overt hepatic encephalopathy (oHE), occurring in 35–50% of patients (European Association for the Study of the Liver 2022; Ehrenbauer et al. 2023). While the occurrence of HE after TIPS has not been linked to an increased mortality (Nardelli et al. 2024), even mild cognitive impairment is associated with a reduced quality of life and higher need for hospitalization (Agrawal et al. 2015; Ampuero et al. 2018; Flud and Duarte-Rojo 2019). As a result, oHE is one of the main reasons for TIPS diameter reduction and occlusion (Gairing et al. 2022b; Pereira et al. 2016; Schindler et al. 2020). Thus, a cautious selection of patients, in particular with regard to the risk for HE, is widely considered to be of high importance (European Association for the Study of the Liver 2022). Well-established risk factors for post-TIPS oHE include impaired liver function, a history of oHE, and advanced age (Gairing et al. 2022b). Additionally, a larger stent diameter and portal pressure gradient (PPG) reduction as well as sarcopenia have been found to be associated with a greater risk for post-TIPS oHE (Gairing et al. 2022b). Unfortunately, valid predictive factors remain scarce.

Some studies suggested that the presence of minimal HE (mHE) may serve as a valid predictive marker, while other studies only found numerical differences (Berlioux et al. 2014; Ehrenbauer et al. 2023; Nardelli et al. 2016; Senzolo et al. 2019). However, precise assessment of mHE is time consuming, requires trained personnel, even demands expensive special equipment depending on diagnostic test employed (Gairing et al. 2022b), and may therefore not be suitable to monitor cognitive function after TIPS in routine clinical care. Moreover, the pathological mechanism of HE in general is considered multifactorial in nature, with infections, bleeding and hyperammonaemia being several triggers for its onset (Rose et al. 2020). TIPS insertion adds to the complexity with a sudden increase of portosystemic shunt volume, which by itself is a well-known risk factor for HE (Praktiknjo et al. 2020; Simón-Talero et al. 2018). Via the new shunt, the ammonia-rich blood from the gut bypasses the liver parenchyma, effectively circumventing the urea cycle, resulting in further impaired ammonia elimination (Gairing et al. 2022b). However, this effect may partly be balanced by the benefits of portal pressure reduction including vastly reduced rates of portal hypertensive bleeding, hyponatremia, ascites as well as a mitigation of sarcopenia (García-Pagán et al. 2020; Larrue et al. 2023; Tsien et al. 2013).

An important and so far still incompletely studied component of HE development is systemic inflammation (SI), which is nowadays considered a main driver of disease progression in advanced cirrhosis (Arroyo et al. 2021; Costa et al. 2021; Engelmann et al. 2021). Recent studies documented a synergistic mechanism of SI in the development of oHE. Several systemic inflammatory markers (SIM), including TNF-α and IL-1β, have been associated with blood brain barrier dysfunction and consecutively increased sensitivity to ammonium (Aldridge et al. 2015; de Vires et al. 1996). Some SIM, most prominently IL-6, have been directly linked to impaired cognitive function (Gairing et al. 2022a). Thus, some authors suggested a promising role for SIM in the diagnosis of mHE and the prediction of oHE (Gairing et al. 2022a; Li et al. 2023). However, the relevance and predictive value of SIM for HE in TIPS patients remain vastly unknown. Hence, this study aims to investigate the link between SIM and the presence of mHE as well as the development of oHE after TIPS insertion.

## Methods

A number of 109 prospectively recruited patients undergoing TIPS-insertion at Hannover Medical School between 08/2019 and 02/2022 were considered for this study. As part of the patients’ enrollment in the prospective Hannover TIPS patient registry (Clinical Trials.gov number NCT04801290), patients underwent blood sampling from the cubital vein in addition to psychometric testing both before TIPS (at baseline) and during structured follow-up (FU) at 1, 3, 6 and 12 months after TIPS-insertion.

**Fig. 1:**
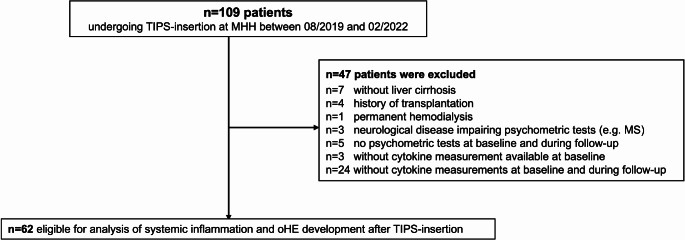
Patient selection and exclusion criteria

Patients without liver cirrhosis and those with history of transplantation, permanent hemodialysis and neurological diseases were excluded, as were patients without psychometric tests or measurement of SIM at baseline. Finally, 62 patients were eligible for analysis of SI and oHE development after TIPS (Fig. 1).

### Data assessment and psychometric testing

Patients were prospectively followed up for death, liver transplantation, as well as oHE development within two years after TIPS insertion. Medication for HE prophylaxis was evaluated at time of psychometric testing before and at discharge after TIPS insertion. Diagnosis for oHE (grade ≥ 2) was based on the West Haven Criteria and ISHEN (International Society for Hepatic Encephalopathy and Nitrogen Metabolism) criteria (Weissenborn 2019). Psychometric testing was performed before TIPS as well as at FU1, FU3, FU6 and FU12. It included the PSE syndrome test (version 2.0; 2020) (Schomerus et al. 1999), yielding the psychometric hepatic encephalopathy score (PHES), and the Animal Naming Test (ANT) (Campagna et al. 2017). The standardized and validated PSE test is widely regarded as the gold standard and was thus used to diagnose mHE (cut-off: −4 points) (Weissenborn 2015) in our study. For the ANT, we considered 23 animals per minute as the cut-off value for the German population, following Labenz et al. (Labenz and Schattenberg 2018).

### TIPS placement

All TIPS implantations were performed by clinically experienced interventional radiologists adhering to the institutional standard operating procedure (Marquardt et al. 2015; Meine et al. 2020). Every patient received a polytetrafluoroethylene-covered stent graft (Viatorr^®^, Gore, Flagstaff, Arizona, AZ, USA) with a stent diameter of either 6 (*n* = 14), 7 (*n* = 1), 8 (*n* = 45) or 10 mm (*n* = 2) under general anaesthesia.

### Collection of samples and soluble inflammatory marker assay

Before TIPS insertion and 1, 3, 6 and 12 months thereafter, blood samples (EDTA plasma) were collected from the patient’s cubital vein. Immediately after obtaining the plasma samples, trained members of staff centrifuged the samples for 10 min at 3000 rotations per minute followed by storage of the plasma supernatants at −80 °C. Utilizing the Luminex based multiplex bead assay (Bio-Plex Pro™ Human Cytokine Assays, catalog no. 12007283, BioRad Laboratories, Hercules, CA), concentrations of SIM in all available samples from patients included in this study were quantified adhering to the manufacturer’s instruction manual. BioPlex Manager™ software was employed for the acquiring of samples. If concentrations in samples measured below the range of detection, the value was imputed as half the limit of quantification divided by two. All analyzed samples had not previously been thawed and samples obtained during active infection were excluded from all analyses (Suppl. Figure 9).

### Study design

Three different analyses were performed for this study.

- Analysis 1: Link of selected SIM (IL-6, TNF-α and IL-1β) with psychometric test results (PHES and ANT) before and after TIPS insertion.

Correlation analyses employing Spearman rho were performed for paired variables (tests and respective SIM) both before TIPS and during follow-up at FU1, FU3, FU6 and FU12. Additionally, levels of SIM were compared between patients with and without mHE at all timepoints using Mann-Whitney U test. Moreover, we analyzed whether the changes of SIM were linked to changes in the psychometric test performances utilizing Chi-squared tests.

- Analysis 2: Predictive value of baseline SIM for the occurrence of oHE after TIPS insertion during two-year follow-up after TIPS.

All 62 patients with SIM levels at baseline were monitored for oHE development during two-year follow-up. Competing risk analysis was then employed to investigate the link between baseline SIM as well as mHE test results and post-TIPS oHE. In a multivariable model we adjusted for FIPS (Freiburg index of post-TIPS survival) and known-risk factors of oHE, i.e. stent diameter and history of oHE. As age is included in the FIPS-Score, this factor was not integrated into the multivariable model. In an additional model, we employed albumin instead of the FIPS-Score to avoid multicollinearity.

- Analysis 3: Predictive value of changes in SIM for the occurrence of oHE during further follow-up.

A number of 32 patients with available SIM measurements at FU1 were included in this analysis and monitored for occurrence of oHE during further follow-up starting at FU1, ending at two-years after TIPS-insertion. The predictive value of both the absolute changes of SIM-levels from baseline to FU1 as well as binary coded increase vs. decrease of SIM were investigated in univariable competing risk analyses. Similarly, we included 49 patients in a second, late-follow-up analysis, comprising SIM levels from either FU3, FU6 or FU12, and investigated the follow-up period after the available late-FU and oHE-development, employing the aforementioned analysis. Multivariable analyses could not be employed due to low numbers of events in both further follow-up time frames, respectively.

### Statistics

All statistical analyses were conducted using IBM SPSS Statistics (version 28, SPSS Inc., Chicago, Illinois, USA), GraphPad Prism (GraphPad Software, Version 5) and R Statistical Software (version 4.2.0. with packages “RCmdr” and “rcmr.EZR plug-in”).

Continuous variables are reported as median, if not indicated otherwise, with interquartile range, and categorical variables as numbers and percentages. For comparisons of continuous variables we utilized the Mann-Whitney U test and analogously for categorical variables the Chi-squared or Fisher’s exact test. Paired t-test were employed to assess statistical differences between paired variables. Further, correlation between parameters was tested using the Spearman rho test. The predictive value of SIM for oHE development during follow-up was tested using the time-dependent Fine-Gray model for competing risk analysis, treating death and liver transplantation as competing events. In order to evaluate the prognostic value of previously proposed IL-6 cut-offs, we employed Tarone-Ware tests due to crossing survival curves, analogous to log-rank tests in the referenced previous study (Li et al. 2023). A p-value < 0.05 was considered statistically significant.

### Ethics

All patients gave written informed consent to enroll in this prospective study, registered at ClinicalTrials.gov (trial number: NCT04801290). The local ethics committee of Hannover Medical School approved this study (protocol identification number: Nr. 8498_BO_S_2019), which was conducted according to the principles of the Declaration of Helsinki.

## Results

### Baseline characteristics of patients

Overall, data of 62 patients with liver cirrhosis were analyzed. The majority of patients were male (64.5%) with a median age of 58 years and median MELD- and FIPS-Scores of 11 and − 0.28, respectively. Most patients (79.0%) were categorized as Child-Pugh B before TIPS-insertion. Alcohol-associated liver disease was the predominant etiology of cirrhosis (51.6%). TIPS-insertion reduced the portal pressure gradient (PPG) from a median of 15mmHg before to a median of 6mmHg, with most common indication being the treatment of refractory ascites (75.8%).

At time of psychometric testing before TIPS-insertion, median PHES was −6 (−10 - −4) and 36 (67.9%) patients presented with mHE as defined by an abnormal PHES. Similarly, the ANT was pathological in 39 (68.4%) patients with a median number of 19 (15–24) animals named. At discharge after TIPS-insertion, 82.3% of patients received HE prophylaxis, most commonly Lactulose (77.4%). All baseline characteristics of the study cohort are displayed in Table 1.

**Table 1:** Baseline characteristics of patients

Baseline characteristics	All patients (*n* = 62)
Age (years)	58.00 [51.25, 65.75]
Sex female/male (%male)	22/40 (64.5%)
Etiology of cirrhosis*	
ALD (%)	32 (51.6%)
MetALD (%)	8 (12.9%)
MASLD (%)	6 (9.7%)
Viral (%)	4 (6.5%)
Cryptogenic (%)	4 (6.5%)
Other (%)	10 (16.1%)
Indication for TIPS*	
Refractory Ascites (%)	47 (75.8%)
Variceal bleeding (%)	21 (33.9%)
Other (%)	1 (1.6%)
PPG before TIPS (mmHg)	15.00 [13.00, 17.75]
PPG after TIPS (mmHg)	6.00 [4.00, 8.00]
**Stent diameter (%)**	
6	14 (22.6%)
7	1 (1.6%)
8	45 (72.6%)
10	2 (3.2%)
MELD (median [IQR])	10.80 [8.57, 13.74]
FIPS (median [IQR])	−0.28 [−0.74, −0.09]
**Child Pugh (%)**	
A	10 (16.1%)
B	49 (79.0%)
C	3 (4.8%)
Bilirubin (mg/dl)	0.91 [0.54, 1.51]
Creatinine (mg/dl)	1.06 [0.86, 1.34]
Sodium (mmol/l)	135.00 [132.00, 137.00]
Platelets (10^3^/µl)	115.00 [76.25, 181.75]
White blood cells (10^3^/µl)	5.10 [3.80, 6.77]
CHE (kU/L)	2.52 [1.86, 4.15]
Albumin (g/l)	30.50 [27.25, 36.00]
AST (U/l)	37.50 [30.25, 47.75]
ALT (U/l)	25.00 [17.00, 33.00]
Ammonia (µmol/l)	47.00 [34.00, 69.50]
CRP (mg/l)	8.90 [3.40, 19.70]
Interleukin-6 (pg/ml)	4.20 [2.32, 8.88]
Tumor Necrosis Factor alpha (TNF-α) (pg/ml)	13.20 [10.33, 17.59]
Interleukin-1 beta (IL-1β) (pg/ml)	1.29 [1.01, 1.83]
Diabetes Mellitus (%)	21 (34.4%)
History of HE (%)	15 (24.2%)
PHES	−6.00 [−10.00, −4.00]
Pathological PHES (%)	36 (67.9%)
ANT-Score	19 [15, 24]
Pathological ANT (%)	39 (68.4%)
Any HE prophylaxis at discharge (%)	51 (82.3%)
Lactulose (%)	48 (77.4%)
Rifaximin (%)	26 (41.9%)
L-ornithine L-aspartate (%)	9 (14.5%)
Lactulose or Rifaximin (%)	51 (82.3%)

* Mixed etiology and indication for TIPS was possible, thus the summation of percentages exceeds 100% in these columns

Mann-Whitney U test was used for continuous variables, shown with median and IQR, and Chi-squared or Fisher’s exact test for categorical variables including percentages

Abbreviations: TIPS: transjugular intrahepatic portosystemic shunt; ALD: alcohol related liver disease; MetALD: metabolic and alcohol related liver disease; MASLD: metabolic dysfunction-associated steatotic liver disease; MELD: model for end-stage liver disease; FIPS: Freiburg index of post-TIPS survival; PPG: portal pressure gradient; CHE: cholinesterase; AST: aspartate aminotransferase; ALT: alanine aminotransferase; CRP: C-reactive protein; HE: hepatic encephalopathy; PHES: psychometric hepatic encephalopathy score; ANT: animal naming test

### Correlation of psychometric test results, mHE and SIM before TIPS insertion

**Fig. 2:**
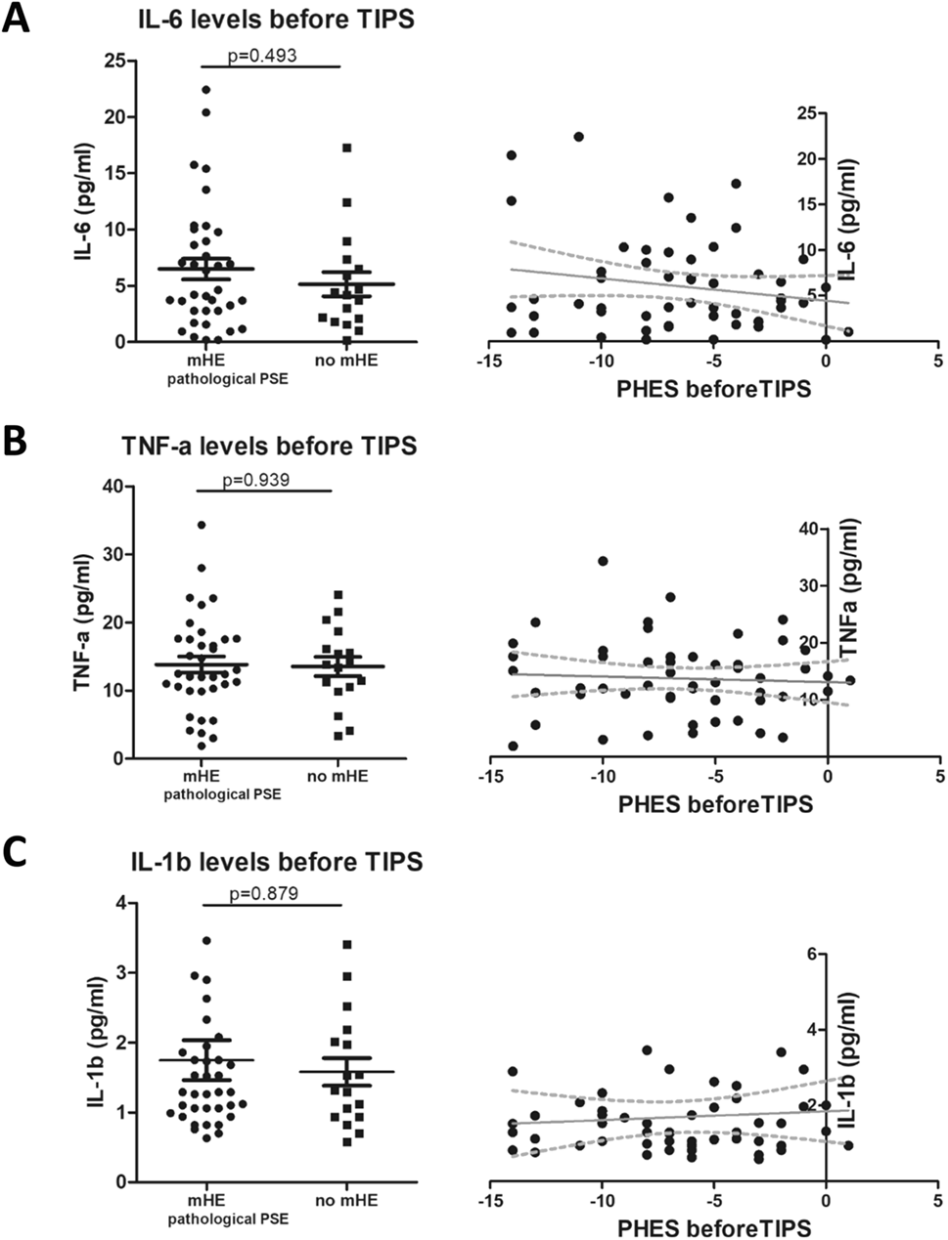
**Comparison of levels of IL-6 (A), TNF-α (B) and IL-1β (C) between patients with mHE and patients without mHE before TIPS and their respecive correlation with PHES.** Figure legend: Shown are levels of investigated SIM (IL-6, TNF-α, IL-1β) stratified according to diagnosis of mHE, compared using Mann-Whitney U test, on the left hand side. A linear regression of obtained PHES and respective SIM is displayed on the right hand side of the paired graphs. Psychometric testing yielding the PHES as well as measurement of SIM were conducted before TIPS-insertion. Abbreviations: TIPS: transjugular intrahepatic portosystemic shunt; mHE: minimal hepatic encephalopathy; PHES: psychometric hepatic encephalopathy score; IL-6: Interleukin 6; TNF-α: Tumor Necrosis Factor alpha; IL-1β: Interleukin 1 beta; SIM: soluble inflammatory markers

The levels of all SIM (IL-6, TNF-α and IL-1β) before TIPS stratified according to mHE or no mHE at psychometric testing before TIPS were comparable (mHE vs. no mHE: IL-6 4.42 vs. 4.2 pg/ml, *p* = 0.493; TNF-α 12.46 vs. 13.82 pg/ml, *p* = 0.939; IL-1β 1.29 vs. 1.32 pg/ml, *p* = 0.879) (Fig. 2, Suppl. Table 1). Likewise, none of the investigated SIM showed any statistically significant correlation with PHES or ANT before TIPS (PHES: IL-6 −0.87, *p* = 0.53; TNF-α −0.06, *p* = 0.66, IL-1β 0.02, *p* = 0.91; ANT: IL-6 −0.03, *p* = 0.80, TNF-α 0.02, *p* = 0.88, IL-1β 0.10, *p* = 0.45) (Table 2).

**Table 2:** Spearman rho correlations of PHES and ANT with SIM before TIPS-insertion (baseline) and during follow-up

**Baseline**		**IL-6**	**TNF-α**	**IL-1β**
PHES	Correlation coefficient	−0.87	−0.063	0.016
	*p*-value	0.534	0.656	0.912
	Number of patients	53	53	53
ANT	Correlation coefficient	−0.34	0.020	0.103
	*p*-value	0.801	0.881	0.446
	Number of patients	57	57	57
**FU1**		**IL-6**	**TNF-α**	**IL-1β**
PHES	Correlation coefficient	0.193	0.352	0.046
	*p*-value	0.306	0.057	0.810
	Number of patients	30	30	30
ANT	Correlation coefficient	0.015	0.126	−0.43
	*p*-value	0.939	0.514	0.826
	Number of patients	29	29	29
**FU3**		**IL-6**	**TNF-α**	**IL-1β**
PHES	Correlation coefficient	−0.014	0.092	0.006
	*p*-value	0.932	0.562	0.967
	Number of patients	42	42	42
ANT	Correlation coefficient	−0.056	−0.093	0.156
	*p*-value	0.726	0.556	0.325
	Number of patients	42	42	42
**FU6**		**IL-6**	**TNF-α**	**IL-1β**
PHES	Correlation coefficient	−0.274	0.302	−0.063
	*p*-value	0.184	0.143	0.764
	Number of patients	25	25	25
ANT	Correlation coefficient	−0.107	0.005	0.039
	*p*-value	0.604	0.980	0.849
	Number of patients	26	26	26
**FU12**		**IL-6**	**TNF-α**	**IL-1β**
PHES	Correlation coefficient	0.103	0.248	−0.131
	*p*-value	0.738	0.415	0.669
	Number of patients	13	13	13
ANT	Correlation coefficient	−0.208	0.466	0.119
	*p*-value	0.495	0.108	0.700
	Number of patients	13	13	13

Abbreviations: TIPS: transjugular intrahepatic portosystemic shunt; PHES: psychometric hepatic encephalopathy score; ANT: animal naming test; FU: follow-up; IL-6: Interleukin 6; TNF-α: Tumor Necrosis Factor alpha; IL-1β: Interleukin 1 beta

**Fig. 3:**
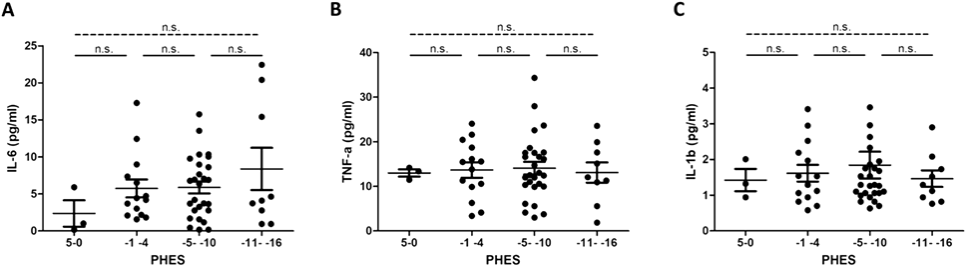
**Comparison of PHES and levels of IL-6 (A), TNF-α (B) and IL-1β (C) before TIPS insertion.** Figure legend: Shown are levels of investigated SIM levels (IL-6, TNF-α, IL-1β) stratified according to PHES at time of psychometric testing before TIPS insertion. For comparison of SIM levels Mann-Whitney U test were performed. Abbreviations: TIPS: transjugular intrahepatic portosystemic shunt; PHES: psychometric hepatic encephalopathy score; IL-6: Interleukin 6; TNF-α: Tumor Necrosis Factor alpha; IL-1β: Interleukin 1 beta; n.s.: not statistically significant *p* > 0.05); SIM: soluble inflammatory markers

Stratification of PHES in subgroups revealed numerically higher IL-6 in patients with highly pathological scores (−11- −16: IL-6 8.37 pg/ml) as compared to those with either mildly pathological (-5- -10: IL-6 5.86 pg/ml) or normal test results (IL-6 5.14 pg/ml). The levels of IL-1β and TNF-α did not show any differences across PHES (Fig. 3, Suppl. Table 2). Treatment with Rifaximin and Lactulose was not found to impact SIM levels (Suppl. Figure 7).

Two previously proposed cut-offs of IL-6 > 7 or IL-6 > 8 pg/ml for stratification of patients with mHE and without likely mHE yielded a sensitivity of 36.1% and 30.6% as well as a specificity of 76.5% and 82.4%, respectively, in our cohort (Suppl. Table 3).

### Correlation of psychometric test results, mHE and SIM after TIPS

**Fig. 4:**
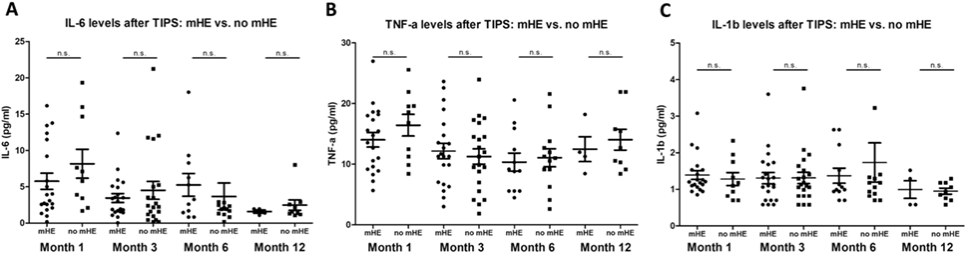
**Comparison of levels of IL-6 (A), TNF-α (B) and IL-1β (C) between patients with mHE and patients without mHE during follow-up after TIPS.** Figure legend: Shown are levels of investigated SIM (IL-6, TNF-α, IL-1β), stratified according to diagnosis of mHE, at follow-up 1, 3, 6 and 12 months after TIPS-insertion. For group comparisons we used Mann-Whitney-U tests. To improve readability of the figures, two outliers were not pictured (IL-6 no mHE group at FU6 = 25.84pg/ml, and IL-1β, same group = 7.8pg/ml). Abbreviations: TIPS: transjugular intrahepatic portosystemic shunt; mHE: minimal hepatic encephalopathy; IL-6: Interleukin 6; TNF-α: Tumor Necrosis Factor alpha; IL-1β: Interleukin 1 beta; n.s.: not statistically significant; SIM: soluble inflammatory markers

Both PHES and ANT results showed statistically significant improvements from baseline to FU12 (median PHES − 6 vs. −2.5, *p* = 0.007; median ANT 19 vs. 23, *p* = 0.004) (Suppl. Figure 1). Similarly, the percentage of pathological test results obtained during follow-up declined (67.9–38.2%, *p* = 0.006; Table 3). When comparing the levels of IL-6, TNF-α and IL-1β between patients with mHE and those without at the respective follow-ups, no statistically significant differences between the considered groups became apparent at any time point (Fig. 4). Similarly, correlation analyses of the investigated SIM at the respective follow-ups with the corresponding PHES or ANT did not yield statistically significant results at any of the follow-ups (Table 2, Suppl. Figure 2). Co-medication did not impact the levels of SIM at any time point, except for lower levels of TNF-α at FU3 in patients treated with Rifaximin (7.92 vs. 13.07, *p* = 0.003, Suppl. Figure 8), which was attributable to a lower FU3 MELD in this group (10 vs. 15).

**Table 3:** Results of mHE-tests before TIPS-insertion and during follow-up

	Baseline (before TIPS)	FU1 (1 Month)	FU3 (3 Months)	FU6 (6 Months)	FU12 (12 Months)
PHES (IQR)	−6 (−10, −3.50)	−7 (−10.75, −3.25)	−4 (−9.5, −2.0)	−5 (−9, −3)	−2.5 (−7, 0)
Pathological PHES (%)	36 (67.9%)	31 (64.6%)	22 (48.9%)	19 (57.6%)	13 (38.2%)
ANT (IQR)	19 (15, 24.5)	19 (14.75,24.5)	20 (15, 27.5)	22 (15.75, 30.25)	23 (18, 27)
Pathological ANT (%)	39 (68.4%)	32 (64.0%)	24 (53.3%)	19 (55.9%)	17 (48.6%)

Abbreviations: TIPS: transjugular intrahepatic portosystemic shunt; PHES: psychometric hepatic encephalopathy score; ANT: animal naming test

**Table 4:** Uni- and multivariable competing risk analysis of development of post-TIPS oHE

	*n*	Univariable analysis		Multivariable analysis	
		HR (95% CI)	*p*-value	HR (95% CI)	*p*-value
IL-6	*62*	1.022 (0.943–1.109)	0.59	1.020 (0.927–1.123)	0.68
TNF-α	*62*	0.994 (0.930–1.062)	0.85	-	
IL-1β	*62*	0.750 (0.503–1.118)	0.16	-	
Age	*62*	1.004 (0.961–1.050)	0.85	-	
FIPS	*62*	2.132 (1.099–4.135)	**0.025**	1.988 (0.871–4.539)	0.10
MELD	*62*	1.022 (0.903–1.157)	0.73	-	
TIPS-indication RA	*62*	0.547 (0.204–1.466)	0.23	-	
Stent diameter (mm)	*62*	1.496 (1.028–2.176)	**0.035**	1.391 (0.902–2.145)	0.14
PPG post TIPS (mmHg)	*62*	0.840 (0.646–1.094)	0.2	-	
CHE (kU/l)	*59*	1.015 (0.751–1.372)	0.92	-	
Albumin (g/l)	*62*	0.905 (0.848–0.965)	**0.002**		
History of oHE	*62*	1.387 (0.472–4.070)	0.55	1.266 (0.389–4.117)	0.69
Any HE prophylaxis	*62*	0.477 (0.176–1.288)	0.14	-	
PHES before TIPS	*53*	0.904 (0.805–1.014)	0.085	-	
Pathological PHES	*53*	1.713(0.573–5.098)	0.33	-	
ANT before TIPS	*57*	0.939 (0.880–1.003)	0.063	-	
Pathological ANT	*57*	1.792 (0.600–5.367)	0.3	-	

Time-dependent Fine–Gray model for competing risk analysis with death or liver transplantation as competing events. 19 (30.6%) patients developed oHE during two-year follow-up. *N* = 62 patients were included in multivariable model, in which we adjusted for FIPS and known risk-factors for oHE. As age and albumin are components of the FIPS-Score, they were not included in the multivariable model despite the first being a known-risk factor for oHE

Values of *p* < 0.05 are highlighted in bold font

Abbreviations: TIPS: transjugular intrahepatic portosystemic shunt; IL-6: Interleukin 6; TNF-α: Tumor Necrosis Factor alpha; IL-1β: Interleukin 1 beta; FIPS: Freiburg index of post-TIPS survival; MELD: model for end-stage liver disease; RA: refractory ascites; PPG: portal pressure gradient; CHE: cholinesterase; oHE: overt hepatic encephalopathy; PHES: psychometric hepatic encephalopathy score; ANT: Animal naming test

Finally, we tested whether a numerical increase of SIM levels between baseline and FU1 or a late FU (3–12 months) after TIPS was associated with a corresponding decrease in PHES and found no significant link (Suppl. Figures 3 and 4).

### Predictive value of SIM before TIPS and during follow-up for development of oHE

During two-year follow-up after TIPS-insertion, 19 (30.6%) patients developed an oHE after a median time of 21 days. In univariable competing risk analysis, FIPS (HR 2.13, *p* = 0.025), albumin (HR 0.905, *p* = 0.002), and larger TIPS-stent-diameter (HR 1.50, *p* = 0.04) were associated with oHE, while baseline IL-6, TNF-α and IL-1β were not significantly linked with oHE (Table 4, Suppl. Table 7).

Previously suggested cut-offs of IL-6 > 12.75 pg/ml as well as IL-6 > 10.5 pg/ml before TIPS did not show a statistically significant stratification of patients with a higher cumulative incidence of oHE in follow-up (Tarone-Ware test *p* = 0.794 and *p* = 0.814, respectively) (Suppl. Figure 5).

All investigated SIM showed a statistically significant decrease during follow-up (Suppl. Figure 6).

In order to evaluate whether a decrease of SIM is linked with oHE, we then analyzed oHE development both after FU1 and after late FU (3–12 months) with consecutive follow-up, respectively. 6/32 (18.8%) patients developed an oHE after FU1 and 6/49 (12.2%) after late FU with a median time to oHE of 113 and 63 days, respectively. Only albumin levels at the respective follow-ups, but neither IL-6-, TNF-α, IL-1β-increase or PHES decrease from pre-TIPS to FU1 or late FU nor MELD and FIPS at the corresponding FU showed significant association with oHE during further follow-up (Suppl. Tables 5, 6).

## Discussion

The occurrence of overt HE represents a clinically relevant and highly prevalent complication after TIPS-insertion. Psychometric tests for diagnosis of mHE have been proposed as predictor of oHE, but this remains controversial (Berlioux et al. 2014; Ehrenbauer et al. 2023; Nardelli et al. 2016). Lately, the role of systemic inflammation in the pathogenesis of HE has gained increasing recognition (Aldridge et al. 2015; Arroyo et al. 2021). As patients treated with a TIPS are generally characterized by a highly progressed liver disease stage, which has been demonstrated to be accompanied by severe systemic inflammation (Arroyo et al. 2021; Costa et al. 2021; Engelmann et al. 2021), we considered the potential relationship between SIM and post-TIPS HE to be of particular interest. Thus, this study investigated the link between psychometric tests and inflammatory markers (SIM) both before and during follow-up after TIPS as well as their predictive value for post-TIPS-oHE.

In the multifactorial pathogenesis of HE, several studies have demonstrated a link between SI and the development of HE (Gairing et al. 2022a, 2022b; Labenz et al. 2019; Shawcross, D. L. et al., 2011). Most importantly, inflammation is thought to render the brain more susceptible to hyperammonemia by impairing the blood-brain barrier (Aldridge et al. 2015; de Vires et al. 1996; Shawcross, Debbie L. et al., 2004; Shawcross, D. L. et al., 2007). In-vitro studies have shown a direct disruption of the barrier integrity by IL-6, TNF-α and IL-1β (de Vires et al. 1996), which is why these particular SIM were selected for the current study. However, TNF-α and IL-1β have not been investigated in this context in clinical setting, so far. Our findings indicate that the effect of these SIM may either not translate from in-vitro to patients with cirrhosis, or the impact of TNF-α and IL-1β could be limited to their secretion by astroglial cells (Aldridge et al. 2015; de Vires et al. 1996) and thus not translate to measurably different levels in cubital vein blood. However, our data regarding IL-6 are in contrast to two previously published studies. In concordance with the aforementioned in-vitro data, Shawcross et al. (2007) and Gairing et al. (2022a ) found significantly higher levels of IL-6 in patients with mHE compared to those without (Gairing et al. 2022a; Shawcross, D. L. et al., 2007). We were not able to replicate the above findings and documented only numerically higher IL-6 levels when stratifying for PHES before TIPS-insertion. This may be attributed to our patients’ characteristics. The study of Gairing et al. included predominantly patients with compensated, Child-Pugh A cirrhosis. The majority of the study cohort published by Shawcross and colleagues had only mild or no ascites. In contrast, almost all of our patients had CHILD B cirrhosis and the far majority were even diagnosed with refractory ascites, which is considered as the advanced, final stage of cirrhosis according to recent classification (D’Amico et al. 2018). Of note, this final stage of cirrhosis is characterized by a high level of systemic inflammation, per se (Costa et al. 2021). In this state, SIM might be of lower value for the prediction of individual cirrhosis associated complications, particularly for the complex and dynamic syndrome of HE. Compared to earlier stages of liver disease, other factors could be of higher relevance for its pathogenesis, i.e. albumin, which we found to be significantly associated with post-TIPS oHE in all employed statistical models.

During follow-up after TIPS-insertion, the levels of investigated SIM also did not correlate with the presence of mHE or psychometric test results. However, diagnosis of mHE by psychometric testing is only validated for HE type C (European Association for the Study of the Liver 2022), characterized by a combination of Type A (reduced detoxification) and B (bypassing of detoxification through portosystemic shunt) (Dharel and Bajaj 2015). It has to be acknowledged that the pathomechanism of HE might change after TIPS insertion. The sudden onset of a large portosystemic shunt might in this setting be of higher importance, similar to a type B rather than a type C HE (Praktiknjo et al. 2020; Simón-Talero et al. 2018). This hypothesis provides an explanation for the particularly high incidence of oHE in the first month after TIPS, the time most influenced by the new onset of shunting. The statistically significant direct link between larger stent diameter and risk for post-TIPS HE, which we also demonstrated in our study, further supports this hypothesis (Schindler et al. 2020). Additionally, in lengthy univariable analyses of all SIM, tests, and known risk-factors for oHE, only portosystemic pressure gradient post TIPS (besides albumin) emerged as a statistically relevant risk factor for oHE development after FU1, further indicating that the shunting may be of higher importance for the pathogenesis of post-TIPS oHE than systemic inflammation.

Acknowledging the growing recognition of the role of systemic inflammation in the pathogenesis of HE, a recent investigation by Li et al. (2023) found a link between high IL-6 levels pre-TIPS and the development of post-TIPS oHE (Li et al. 2023). Participants with IL-6 > 10.5 pg/ml showed a higher incidence of oHE, and a proposed cut-off of 12.75 pg/ml exhibited higher predictive power than other implemented indicators. We did not replicate these findings in our study, most likely due to the significant disparities of patients’ characteristics, particularly regarding the etiology (majority viral) and indication for TIPS (85% variceal bleeding). As nowadays the majority of TIPS are placed for treatment of refractory ascites (Steib et al. 2020), our study provides a suitable representation of real-world-setting. Our deviating results underscore the necessity for further research to validate the efficacy of the predictive and diagnostic properties of individual SIM, and emphasize caution regarding the clinical application of SIM.

Systemic inflammation is understood to predominantly result from bacterial translocation from the intestinal lumen due to portal hypertension (Arroyo et al. 2021; Reiberger et al. 2013). Thus, TIPS-insertion itself may ameliorate the systemic inflammation as a result of portal pressure reduction. Some studies have shown a reduction of selected SIM, such as CXCL10, following TIPS (Lehmann et al. 2018). The investigated SIM IL-6, TNF-α and IL-1β in our study collectively show a steady decrease following TIPS-insertion, thereby suggesting an effect of TIPS on systemic inflammation. However, SIM changes did not correlate with changes in the psychometric tests or oHE development. This may be attributable to the aforementioned rather complex multifactorial pathomechanism of post-TIPS HE and the decreasing cohort size during further follow-up due to the high mortality in patients with decompensated cirrhosis.

Our study has some limitations. Firstly, the above-mentioned relatively small sample size in our study may have translated to a lack of statistical power to detect smaller differences regarding the predictive value of SIM or the correlation of tests and SIM. Secondly, we excluded patients with active infections and hemodialysis before TIPS and during follow-up, thus limiting the generalizability of our results. However, these comorbidities can lead to cognitive deficits per se, which may affect psychometric tests and may not be distinguishable from hepatic encephalopathy. Lastly, despite providing a hypothesis for a reduced risk for post-TIPS HE as a result of a TIPS-mediated amelioration of investigated SIM, this study was not designed to explore the pathomechanism of post-TIPS HE and provide reasoning as to why the incidence of HE post TIPS is elevated despite reduced systemic inflammation. The exploration of this matter remains of high interest for further studies.

In conclusion, our study did not demonstrate a significant link between IL-6, TNF-α and IL-1β and psychometric tests before TIPS and during follow-up thereafter. We found no predictive value of pre-TIPS SIM levels, but a significant decrease of systemic inflammation after TIPS. However, this did not translate to a reduced risk for post-TIPS oHE. Hence, the pathomechanism and predictors for post-TIPS HE remain of particular interest for future studies.

## Electronic supplementary material

Below is the link to the electronic supplementary material.

Supplementary Material 1

## Data Availability

Anonymised data of this study are available from the corresponding author upon reasonable request.

## Abbreviations

ANT: animal naming test
ALD: alcohol related liver disease
CHE: cholinesterase
FIPS: Freiburg index of post-TIPS survival
FU: follow-up after TIPS
HE: hepatic encephalopathy
HR: hazard ratio
IL-6: Interleukin 6
IL-1β: Interleukin 1-beta
ISHEN: International Society for Hepatic Encephalopathy and Nitrogen Metabolism
MASLD: metabolic dysfunction-associated steatotic liver disease
MELD: model for end-stage liver disease
MetALD: metabolic and alcohol related liver disease
mHE: minimal hepatic encephalopathy
oHE: overt hepatic encephalopathy
PHES: psychometric hepatic encephalopathy score
PSE: portosystemic encephalopathy syndrome test
PPG: portal pressure gradient
TIPS: transjugular intrahepatic portosystemic shunt
TNF-α: Tumor Necrosis Factor alpha
SIM: soluble inflammatory marker
