# Supplementary material for: Value of systemic inflammation markers for the detection of minimal and prediction of overt hepatic encephalopathy after TIPS insertion
Source: Metab Brain Dis. 2024 Dec 10;40(1):58. doi: 10.1007/s11011-024-01436-2 (PMC11632008; doi:10.1007/s11011-024-01436-2)
Supplement: Supplementary file 1 — Supplementary Material 1 [file 11011_2024_1436_MOESM1_ESM.docx]

# **Supplementary Material: Tables**

**Suppl. Table 1: Comparison of SIM-levels between patients with mHE and those without before TIPS-insertion** (addition to Fig. 2)

| n=53 | **no mHE*** (n=17) | **mHE*** (n=36) | **p-value** |
| --- | --- | --- | --- |
| **IL-6** in pg/ml (IQR) | 4.2 (1.96-6.94) | 4.42 (2.78-9.57) | 0.493 |
| **TNF-a** in pg/ml (IQR) | 13.82 (10.22-17.41) | 12.46 (10.04-17.59) | 0.939 |
| **IL-1b** in pg/ml (IQR) | 1.32 (0.94-2.10) | 1.29 (1.01-1.83) | 0.879 |

*Diagnosis of mHE was based on abnormal PHES.

**Abbreviations:** SIM: soluble inflammatory marker; mHE: minimal hepatic encephalopathy; IL-6: Interleukin 6

**Suppl. Table 2: SIM levels before TIPS stratified by PHES** (addition to Fig. 4)

| **PHES** total n=53 | **5** to **0** (n=3) | **-1** to **-4** (n=14) | **-5** to **-10** (n=27) | **-11** to **-18** (n=9) |
| --- | --- | --- | --- | --- |
| **IL-6** in pg/ml (IQR) | 2.37 | 5.74 (2.16-7.76) | 5.86 (2.78-8.96) | 8.373 (1.87-17.9) |
| **TNF-a** in pg/ml (IQR) | 13.00 | 13.66 (8.98-19.12) | 14.07 (9.95-17.51) | 13.10 (8.26-18.76) |
| **IL-1b** in pg/ml (IQR) | 1.42 | 1.62 (0.91-2.27) | 1.84 (1.06-1.86) | 1.46 (0.88-1.91) |

Mean levels of SIM stratified by PHES as an addition to Figure 4.

**Abbreviations:** SIM: soluble inflammatory marker; PHES: psychometric hepatic encephalopathy score; IL-6: Interleukin 6

**Suppl. Table 3: Diagnostic accuracy of proposed cut-off of IL-6 >8 (A) and IL-6 >7 (B) pg/ml for detection of minimal hepatic encephalopathy before TIPS insertion**

|  | **Sensitivity** | **Specificity** | **NPV** | **PPV** |
| --- | --- | --- | --- | --- |
| **IL-6 >7 pg/ml** | 36.1% | 76.5% | 36.1% | 76.5% |
| **IL-6 >8 pg/ml** | 30.6% | 82.4% | 35.9% | 73.3% |

**Abbreviations:** IL-6: Interleukin 6; NPV: negative predictive value; PPV: positive predictive value

**Suppl. Table 4: Levels of soluble inflammatory markers (IL-6, TNF-a, IL-1b) before TIPS-insertion and during follow-up** (in addition to Suppl. Fig 3)

|  | **Baseline**  (before TIPS) | **FU1**  **(1 Month)** | **FU3**  **(3 Months)** | **FU6**  **(6 Months)** | **FU12**  **(12 Months)** |
| --- | --- | --- | --- | --- | --- |
| **IL-6 in pg/ml** (IQR) | 4.2  (2.16, 8.97) | 3.9  (2.12, 10.93 | 2.21  (1.39, 4.94) | 2.42  (1.30, 4.33) | 1.48  (1.17, 2.28 |
| **TNF-a in pg/ml**(IQR) | 13.20  (10.21, 17.61) | 14.15  (10.05, 18.61) | 10.28  (6.93, 15.10) | 10.10  (6.84, 14.17) | 11.63  (8.98, 16.39) |
| **IL-1b in pg/ml** (IQR) | 1.29  (0.98, 1.88) | 1.21  (1.01, 1.53) | 1.1  (0.82, 1.55) | 1.06  (0.87, 1.41) | 0.90  (0.58, 1.21) |

**Abbreviations:** TIPS: transjugular intrahepatic portosystemic shunt; IL-6: Interleukin 6; TNF-a: Tumor Necrosis Factor alpha; IL-1b: Interleukin 1 beta; FU: follow-up after TIPS.

*BL: n=62; FU1: n=32; FU3: n=46; FU6: n=34; FU12: n=14*

**Suppl. Table 5: Univariable competing risk analysis of development of post-TIPS oHE after FU1**

|  | *n* | **Univariable analysis** | |
| --- | --- | --- | --- |
|  |  | **HR (95% CI)** | **p-value** |
| IL-6 | *32* | 1.027 (0.913-1.155) | 0.66 |
| IL-6 increase to FU1 | *32* | 2.462 (0.489-12.39) | 0.27 |
| ∆ IL-6 (FU1-BL) | *32* | 1.052 (0.952-1.163) | 0.32 |
| TNF-a | *32* | 1.047 (0.961-1.141) | 0.29 |
| TNF-a increase to FU1 | *32* | 1.219 (0.261-5.693) | 0.8 |
| ∆ TNF-a (FU1-BL) | *32* | 1.006 (0.869-1.164) | 0.94 |
| IL-1b | *32* | 1.423 (0.576-3.516) | 0.44 |
| IL-1b increase to FU1 | *32* | 3.168 (0.6242-16.08) | 0.16 |
| ∆ IL-1b (FU1-BL) | *32* | 2.164 (0.612-7.653) | 0.23 |
| Age | *32* | 1.008 (0.935-1.087) | 0.83 |
| FIPS FU1 | *31* | 1.184 (0.517-2.713) | 0.69 |
| MELD FU1 | *32* | 1.005 (0.825-1.226) | 0.96 |
| TIPS-indication RA | *32* | 1.750 (0.217-14.09) | 0.60 |
| Stent diameter (mm) | *32* | 1.00 (0.500-2.000) | 1.00 |
| PSG post TIPS (mmHg) | *32* | 0.668 (0.481-0.926) | **0.016** |
| History of HE | *32* | 1.78 (0.322-9.84) | 0.51 |
| PHES FU1 | *30* | 0.829 (0.658-1.045) | 0.11 |
| Pathological PHES | *30* | 2.211 (0.286-0.450) | 0.45 |
| PHES decrease to FU1 | *25* | 1.018 (0.180-5.763) | 0.98 |
| ∆ PHES (FU1-BL) | *25* | 1.024 (0.887-1.182) | 0.75 |
| ANT FU1 | *29* | 0.812 (0.587-1.124) | 0.21 |
| Pathological ANT | *29* | 2.109 (0.240-18.51) | 0.5 |
| Albumin FU1 | *32* | 0.842 (0.727-0.974) | **0.021** |

Time-dependent Fine–Gray model for competing risk analysis with death or liver transplantation as competing events. 6 (18.75%) patients developed oHE after FU1 during two-year follow-up.

Values of p <0.05 are highlighted in bold font.

**Abbreviations:** TIPS: transjugular intrahepatic portosystemic shunt; IL-6: Interleukin 6; TNF-a: Tumor Necrosis Factor alpha; IL-1b: Interleukin 1 beta; FU1: follow-up one month after TIPS; FIPS: Freiburg index of post-TIPS survival; MELD: model for end-stage liver disease; RA: refractory ascites; PPG: portal pressure gradient; CHE: cholinesterase; oHE: overt hepatic encephalopathy; PHES: psychometric hepatic encephalopathy score ; ANT: Animal naming test

**Suppl. Table 6: Univariable competing risk analysis of development of post-TIPS oHE after late FU**

|  | *n* | **Univariable analysis** | |
| --- | --- | --- | --- |
|  |  | **HR (95% CI)** | **p-value** |
| IL-6 | *49* | 1.034 (0.956-0.113) | 0.38 |
| IL-6 increase to lateFU | *49* | 0.800 (0.094-6.775) | 0.84 |
| ∆ IL-6 (lateFU-BL) | *49* | 0.972 (0.861-1.097) | 0.64 |
| TNF-a | *49* | 1.001 (0.864-1.159) | 0.99 |
| TNF-a increase to lateFU |  | 0.508 (0.073-3.523) | 0.49 |
| ∆ TNF-a (late FU-BL) | *49* | 0.956 (0.816-1.120) | 0.58 |
| IL-1b | *49* | 0.716 (0.201-2.549) | 0.61 |
| IL-1b increase to lateFU | *49* | 3.555 (0.782-16.170) | 0.10 |
| ∆ IL-1b (lateFU-BL) | *49* | 2.363 (0.952-5.866) | 0.064 |
| Age | *49* | 1.021 (0.947-1.101) | 0.590 |
| **FIPS lateFU** | *44* | 2.084 (1.007-4.312) | **0.048** |
| MELD lateFU | *48* | 0.927 (0.746-1.152) | 0.49 |
| TIPS-indication RA |  | * |  |
| Stent diameter (mm) | *49* | 0.789 (0.361-1.172) | 0.55 |
| **PSG post TIPS (mmHg)** | *49* | 0.677 (0.528-0.868) | **0.002** |
| History of HE | *49* | 1.72 (0.321-9.217) | 0.53 |
| **PHES lateFU** | *41* | 0.825 (0.691-0.984) | **0.033** |
| Pathological PHES | *41* | 4.888 (0.530-45.12) | 0.16 |
| PHES decrease to lateFU | *39* | 4.935 (0.567-42.94) | 0.15 |
| ∆ PHES (lateFU-BL) | *39* | 0.830 (0.674-1.017) | 0.072 |
| ANT lateFU | *43* | 0.994 (0.913-1.083) | 0.89 |
| Albumin lateFU | *44* | 0.757 (0.634-0.904) | **0.0021** |

*error in R

Time-dependent Fine–Gray model for competing risk analysis with death or liver transplantation as competing events. 6 (12.2%) patients developed oHE after FU1 during two-year follow-up.

Values of p <0.05 are highlighted in bold font.

**Abbreviations:** TIPS: transjugular intrahepatic portosystemic shunt; IL-6: Interleukin 6; TNF-a: Tumor Necrosis Factor alpha; IL-1b: Interleukin 1 beta; FIPS: Freiburg index of post-TIPS survival; lateFU: late follow-up (3,6 or 12 months after TIPS); MELD: model for end-stage liver disease; RA: refractory ascites; PPG: portal pressure gradient; CHE: cholinesterase; oHE: overt hepatic encephalopathy; PHES: psychometric hepatic encephalopathy score ; ANT: Animal naming test

**Suppl. Table 7: Additional multivariable model for competing risk analysis of development of post-TIPS oHE** (in addition to Table 3)

|  | **Multivariable analysis** | |
| --- | --- | --- |
|  | **HR (95% CI)** | **p-value** |
| IL-6 | 0.994 (0.903-1.094) | 0.900 |
| Stent diameter (mm) | 1.579 (1.040-2.395) | **0.032** |
| History of HE | 1.560 (0.511-4.764) | 0.440 |
| Albumin | 0.908 (0.850-0.970) | **0.004** |

Time-dependent Fine–Gray model for competing risk analysis with death or liver transplantation as competing events. Additional multivariable model with Albumin instead of FIPS to avoid collinearity.

Values of p <0.05 are highlighted in bold font.

**Abbreviations:** IL-6: Interleukin 6; HE: hepatic encephalopathy

# **Supplementary Material: Figures**

**Suppl. Figure 1**: Changes in PHES (A) and ANT (B) during follow-up after TIPS-insertion

**
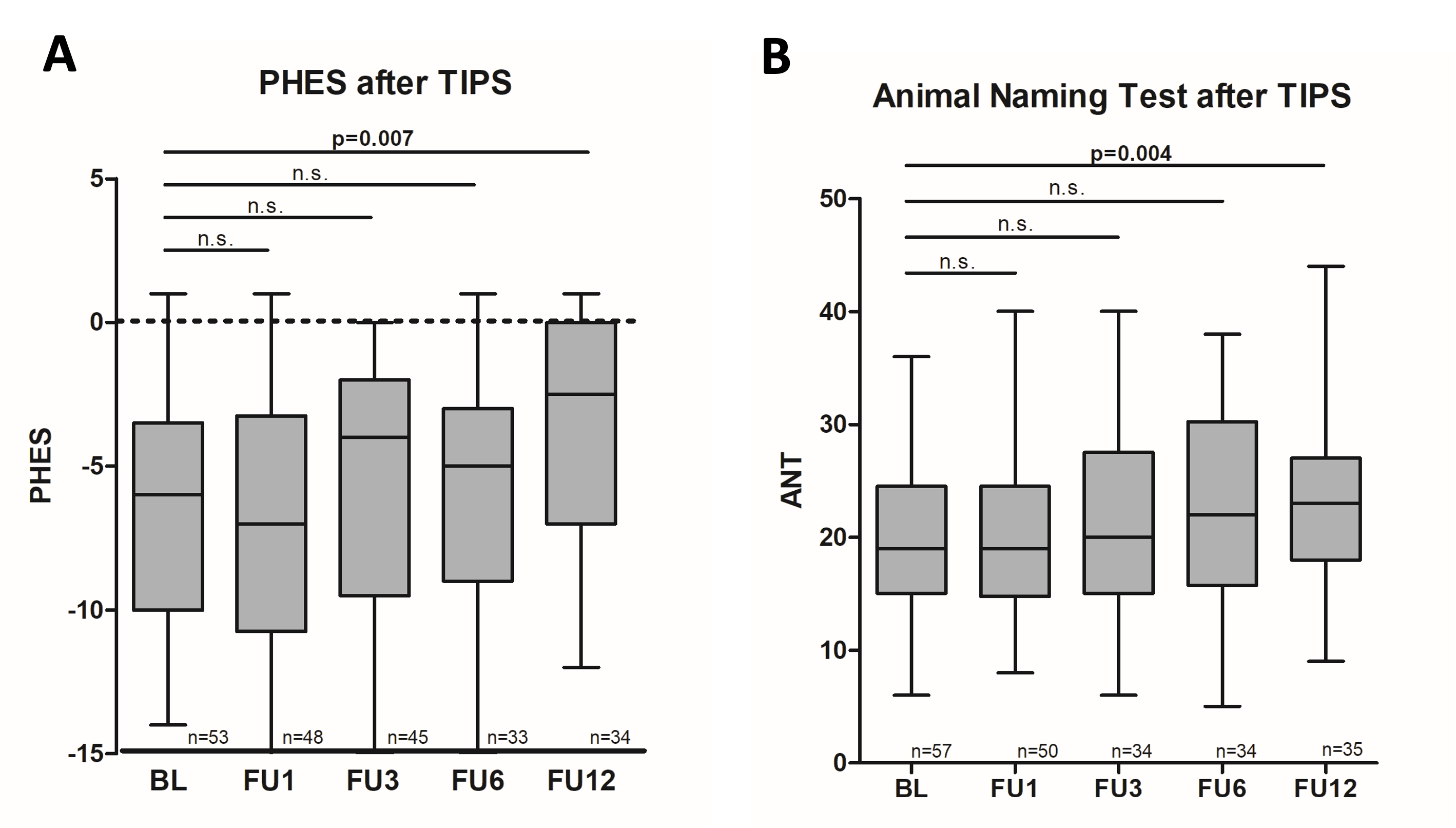
**

**Figure legend:**

Comparison of PHES and ANT before TIPS (BL) and during structured follow-up (FU) 1, 3, 6, and 12 months after TIPS insertion. BL and the respective time-points were compared using paired t-tests.

**Abbreviations:** TIPS: transjugular intrahepatic portosystemic shunt; BL: baseline (before TIPS); FU: follow-up; n.s.: not statistically significant; PHES: psychometric hepatic encephalopathy score; ANT: animal naming test

**Suppl. Figure 2:** Correlation of PHES and SIM during follow-up after TIPS (A: 1 month, B: 3 months, C: 6 months, D: 12 months after TIPS)

**
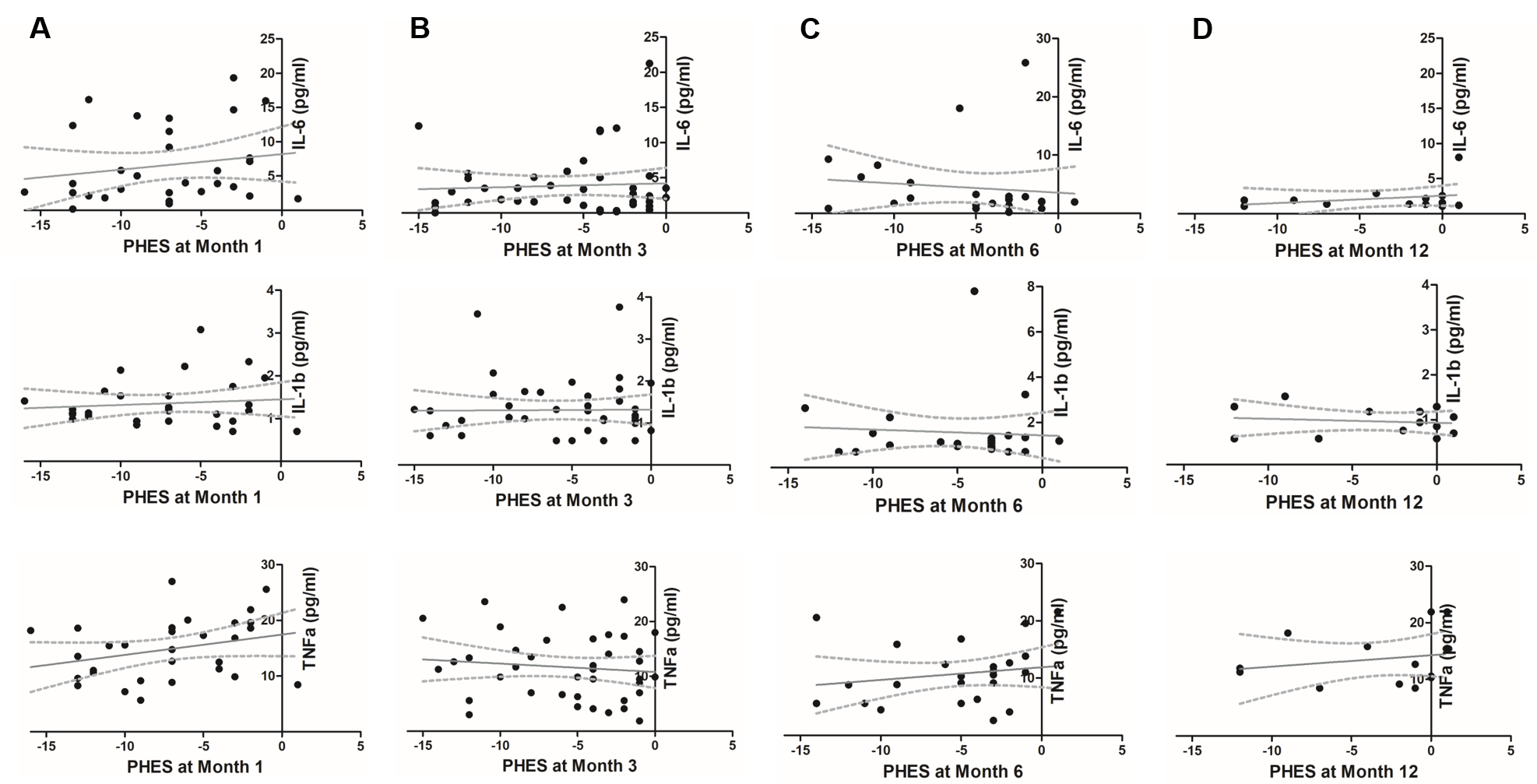
**

**Figure legend:**

Shown are linear regressions of obtained PHES and respective SIM during structured follow-up after TIPS insertion.

**Abbreviations:** TIPS: transjugular intrahepatic portosystemic shunt; mHE: minimal hepatic encephalopathy; PHES: psychometric hepatic encephalopathy score; IL-6: Interleukin 6; TNF-a: Tumor Necrosis Factor alpha; IL-1b: Interleukin 1 beta; SIM: soluble inflammatory markers

**Suppl. Figure 3:** Course of IL-6 (A), TNF-a (B) and IL-1b (C) from before TIPS (BL) to follow-up after 1 Month (FU1) and association of (no) increase with course of PHES during follow-up period

**
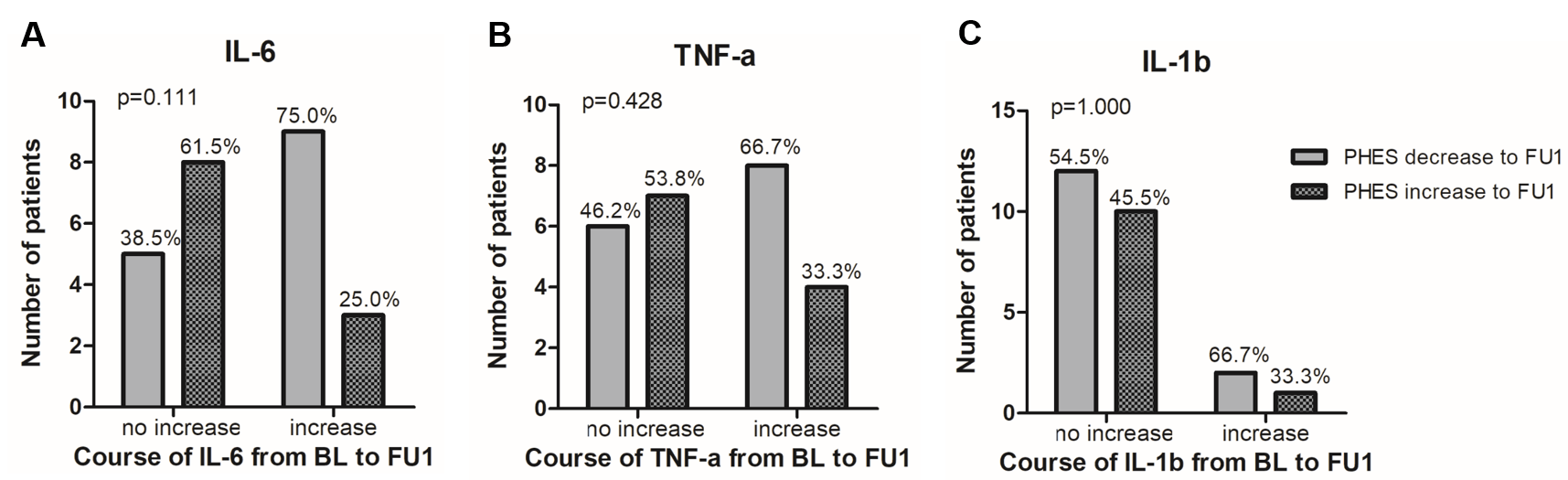
**

**Figure legend:**

Patients at 1 month after TIPS (FU1) were stratified according to the course of SIM (no increase from before TIPS to FU1 vs. Increase). Within the groups we compared the ratio of patients with a decrease or increase of PHES during the same follow-up period, investigating whether there is a link between course of SIM and course of PHES. P-Values were obtained using Chi-Squared tests.

**Abbreviations:** TIPS: transjugular intrahepatic portosystemic shunt; BL: baseline (before TIPS); IL-6: Interleukin 6; TNF-a: Tumor Necrosis Factor alpha; IL-1b: Interleukin 1 beta; FU1: follow-up 1 month after TIPS; PHES: psychometric hepatic encephalopathy score

**Suppl. Figure 4:** Course of IL-6 (A), TNF-a (B) and IL-1b (C) from before TIPS (BL) to late follow-up (late FU) and association of (no) increase with course of PHES during follow-up period

**
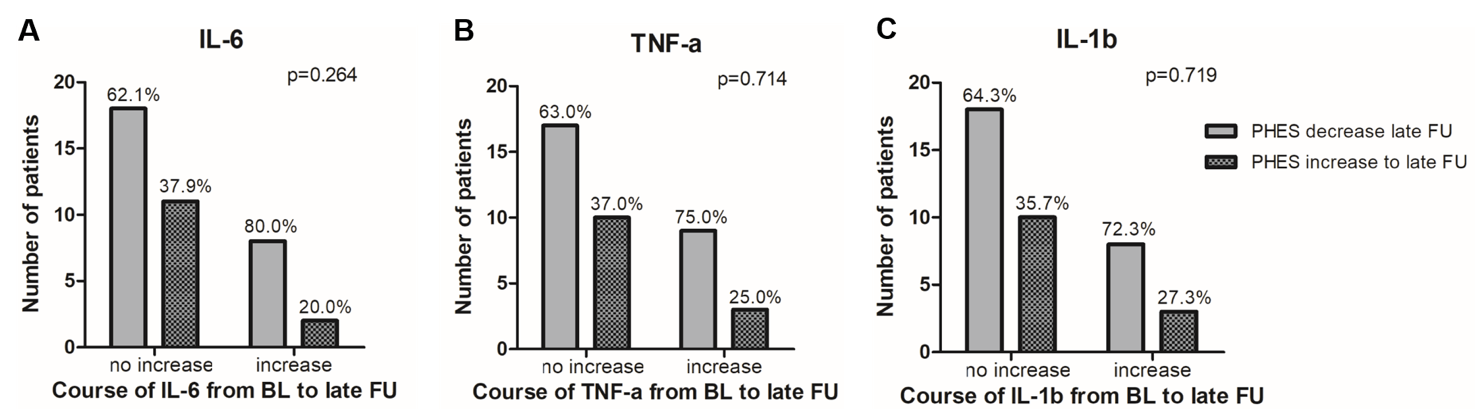
**

**Figure legend:**

Patients at late FU (either 6, 3 or 12 months after TIPS) were stratified according to the course of SIM (no increase from before TIPS to late FU vs. Increase). Within the groups we compared the ratio of patients with a decrease or increase of PHES during the same follow-up period, investigating whether there is a link between course of SIM and course of PHES. P-Values were obtained using Chi-Squared tests.

**Abbreviations:** TIPS: transjugular intrahepatic portosystemic shunt; BL: baseline (before TIPS); IL-6: Interleukin 6; TNF-a: Tumor Necrosis Factor alpha; IL-1b: Interleukin 1 beta; FU: follow-up; PHES: psychometric hepatic encephalopathy score

**Suppl. Figure 5**: Cumulative incidence of post-TIPS oHE stratified by proposed cut-offs of IL-6 >12.75 pg/ml (A) and IL-6 >10.5 pg/ml (B)

**
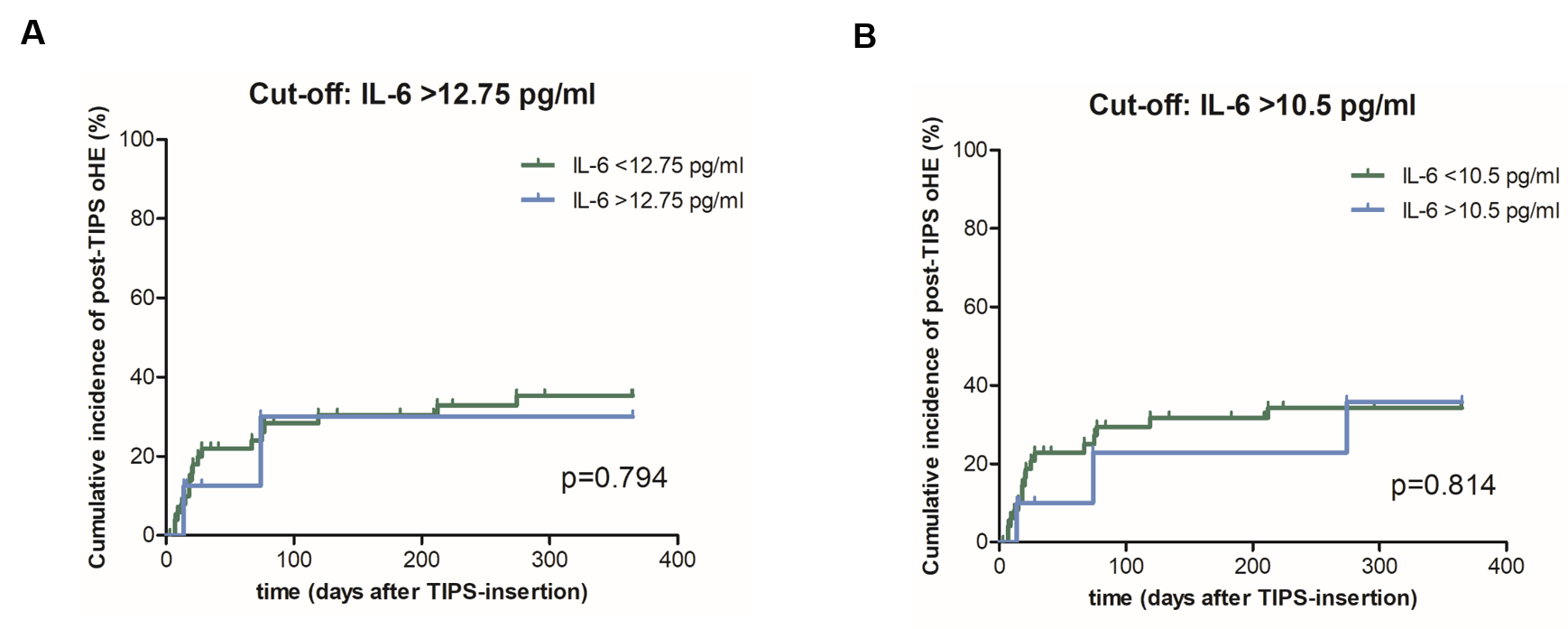
**

**Figure legend:**

Univariable cox regression analysis of previously proposed cut-offs of IL-6>12.75pg/ml and IL-6>10.5pg/ml and development of oHE after TIPS insertion during 356 days of follow-up. P-values were obtained using Tarone-Ware-tests due to intersecting curves.

**Abbreviations:** TIPS: transjugular intrahepatic portosystemic shunt; oHE: overt hepatic encephalopathy; IL-6: Interleukin 6

**Suppl. Figure 6:** Course of SIM after TIPS-insertion

**
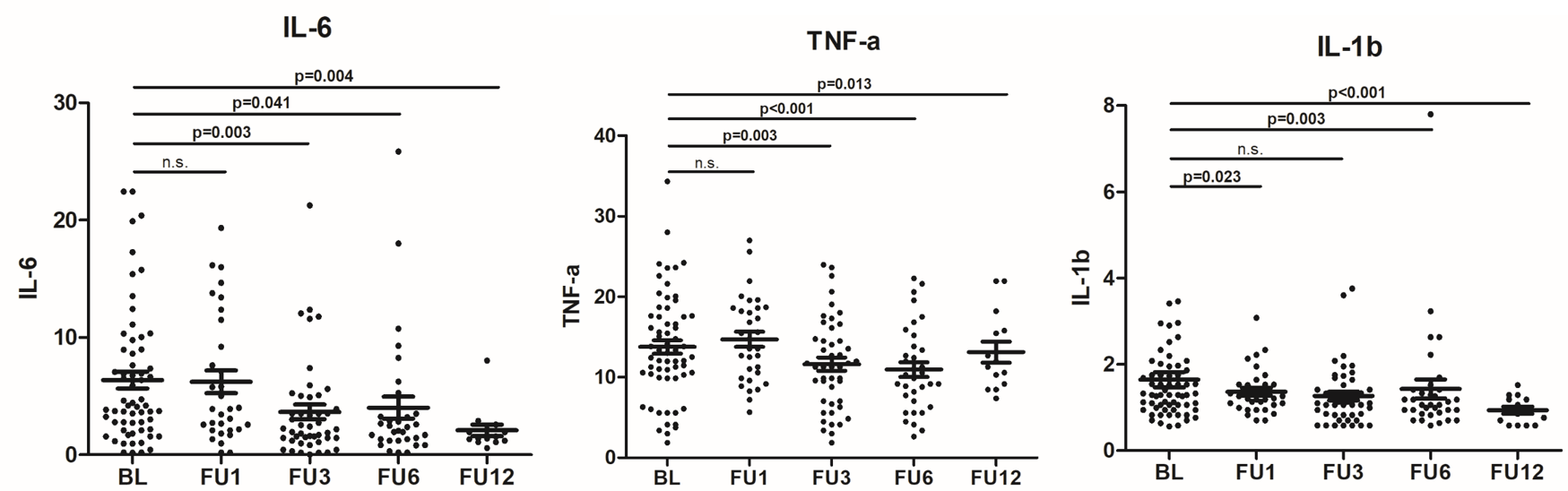
**

**Figure legend:**

Shown are the levels of investigated SIM before TIPS-insertion (BL) and during follow-up at 1, 3, 6, and 12 months after TIPS. Paired t-tests were used to compare SIM between BL and the respective follow-ups.

**Abbreviations:** BL: baseline (before transjugular intrahepatic portosystemic shunt=TIPS); IL-6: Interleukin 6; TNF-a: Tumor Necrosis Factor alpha; IL-1b: Interleukin 1 beta; FU: follow-up; n.s.: not statistically significant

**Suppl. Figure 7:** Impact of co-medication (Lactulose and Rifaximin) on the investigated SIMs at baseline before TIPS


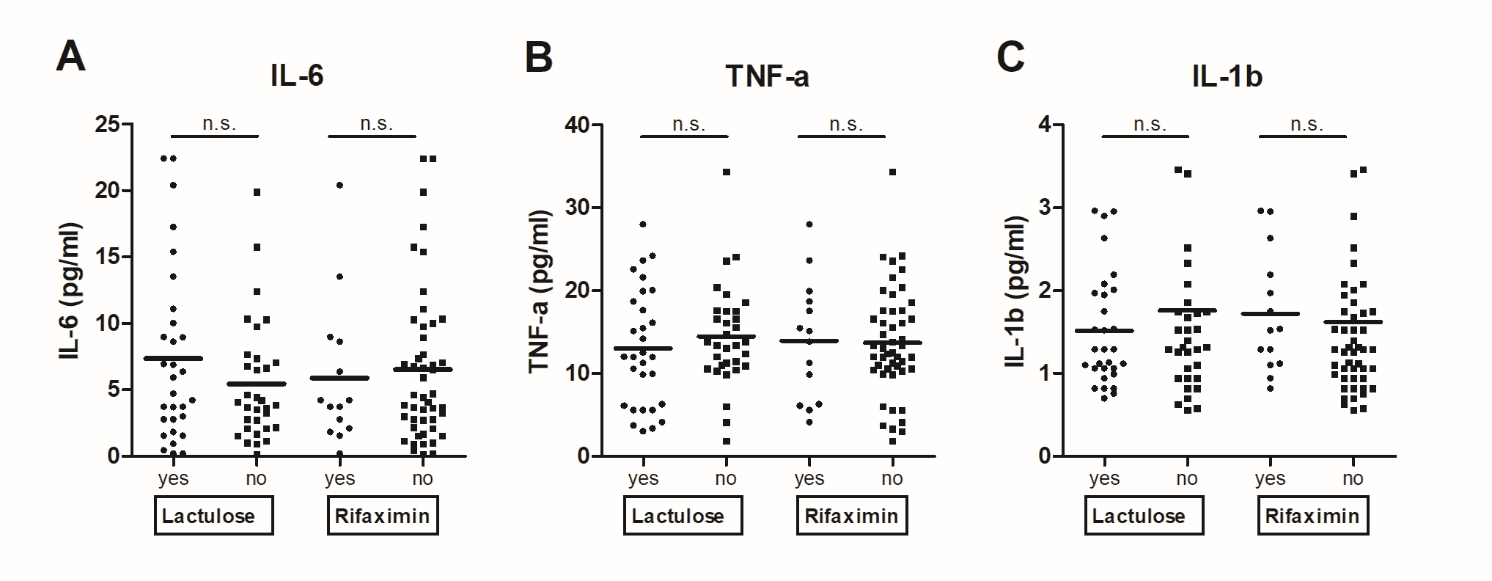


**Figure legend:**

Shown are the levels of investigated SIM before TIPS-insertion (BL) stratified by treatment with Lactulose and Rifaximin. 30 (48.4%) patients received Lactulose, 14 (22.6%) were treated with Rifaximin. Mann-Whitney-U test was used for statistical tests.

**Abbreviations:** IL-6: Interleukin 6; TNF-a: Tumor Necrosis Factor alpha; IL-1b: Interleukin 1 beta; FU: follow-up; n.s.: not statistically significant

**Suppl. Figure 8:** Impact of co-medication (Lactulose and Rifaximin) on the investigated SIMs during follow-up after TIPS

*
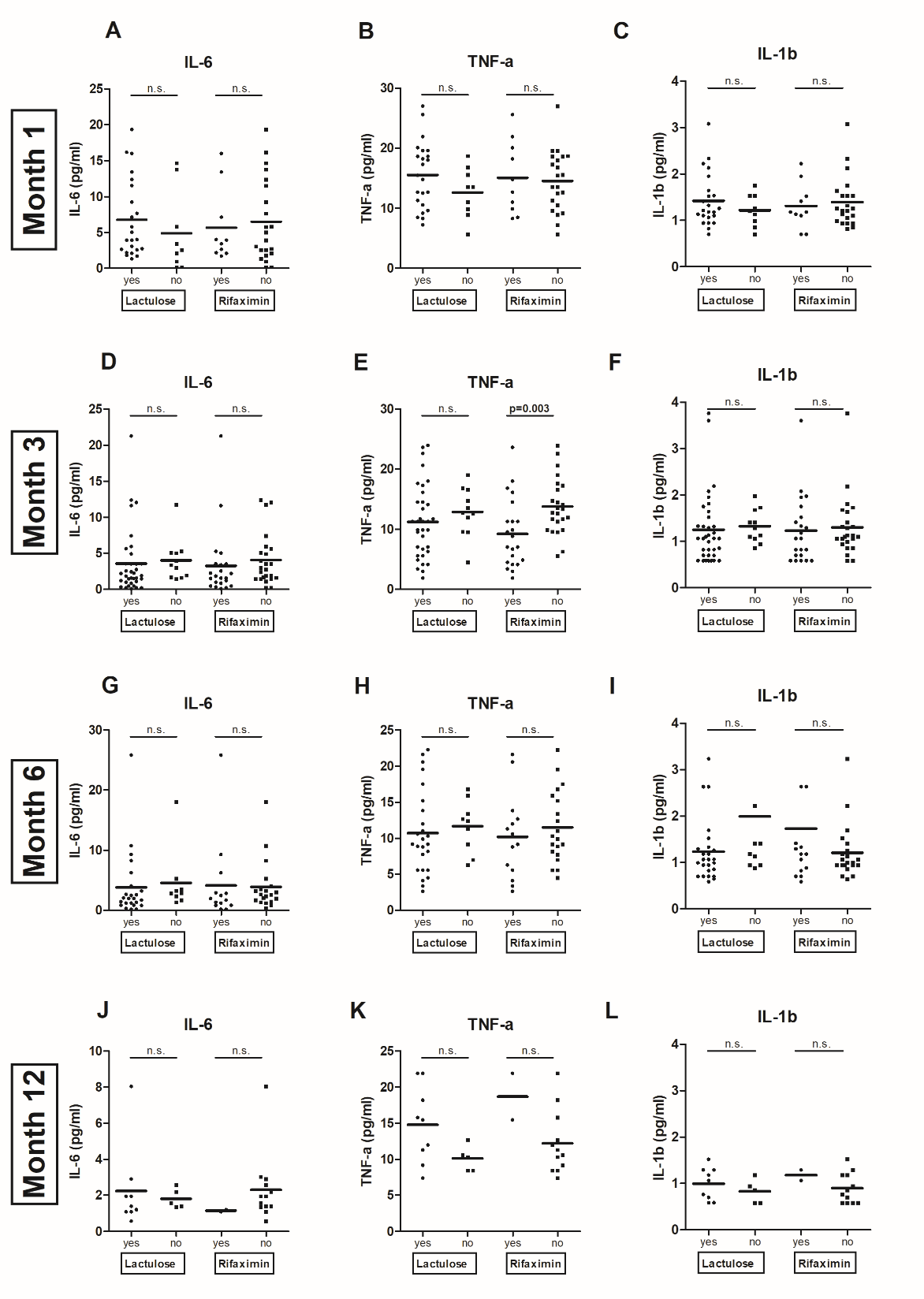
*

**Figure legend:**

Shown are the levels of investigated SIM before TIPS-insertion (BL) stratified by treatment with Lactulose and Rifaximin. 48 (77.4%) patients received Lactulose, 28 (45.2%) were treated with Rifaximin. Mann-Whitney-U test was used for statistical tests.

**Abbreviations:** IL-6: Interleukin 6; TNF-a: Tumor Necrosis Factor alpha; IL-1b: Interleukin 1 beta; FU: follow-up; n.s.: not statistically significant

**Suppl. Figure 9:** Sample selection


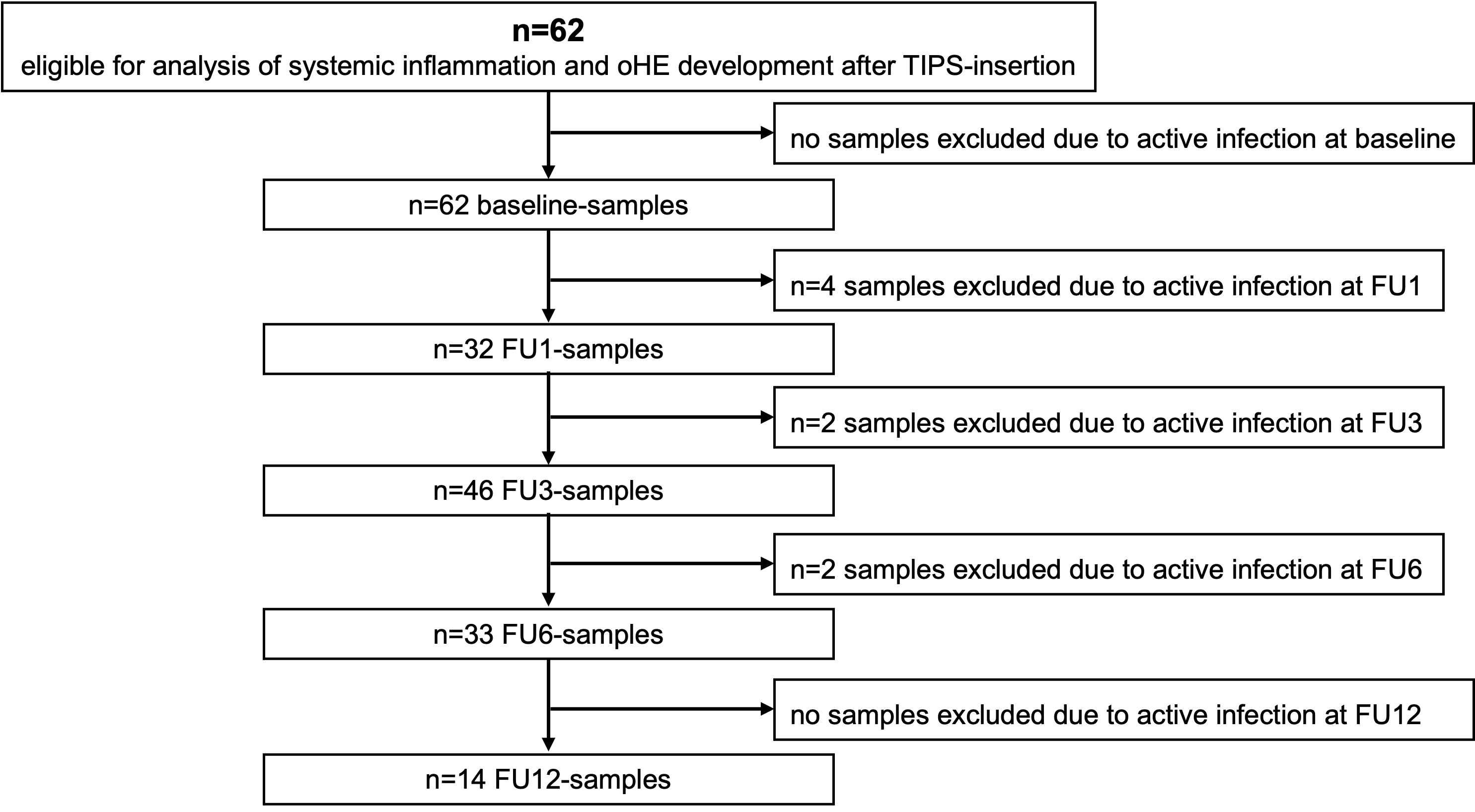


**Figure legend:** Overview of sample selection since samples with active infection at the respective time points were excluded from all analyses.

**Abbreviations:** TIPS, transjugular intrahepatic portosystemic shunt, FU, follow-up (e.g. month after TIPS).
